# Shared Blood Transcriptomic Signatures between Alzheimer’s Disease and Diabetes Mellitus ^†^

**DOI:** 10.3390/biomedicines9010034

**Published:** 2021-01-04

**Authors:** Taesic Lee, Hyunju Lee

**Affiliations:** 1 Department of Biomedical Science and Engineering, Gwangju Institute of Science and Technology, Gwangju 61005, Korea; ddasic123@gist.ac.kr; 2 Artificial Intelligence Graduate School, Gwangju Institute of Science and Technology, Gwangju 61005, Korea; 3 School of Electrical Engineering and Computer Science, Gwangju Institute of Science and Technology, Gwangju 61005, Korea

**Keywords:** Alzheimer’s disease, diabetes mellitus, blood gene expression

## Abstract

Alzheimer’s disease (AD) and diabetes mellitus (DM) are known to have a shared molecular mechanism. We aimed to identify shared blood transcriptomic signatures between AD and DM. Blood expression datasets for each disease were combined and a co-expression network was used to construct modules consisting of genes with similar expression patterns. For each module, a gene regulatory network based on gene expression and protein-protein interactions was established to identify hub genes. We selected one module, where *COPS4*, *PSMA6*, *GTF2B*, *GTF2F2*, and *SSB* were identified as dysregulated transcription factors that were common between AD and DM. These five genes were also differentially co-expressed in disease-related tissues, such as the brain in AD and the pancreas in DM. Our study identified gene modules that were dysregulated in both AD and DM blood samples, which may contribute to reveal common pathophysiology between two diseases.

## 1. Introduction

Alzheimer’s disease (AD), the most common form of dementia, has pathophysiological characteristics that include amyloid plaques, which are extracellular deposits of beta-amyloid (Aβ) in the brain parenchyma; cerebral amyloid angiopathy, which is the accumulation of Aβ in the cerebral blood vessels; and neurofibrillary tangles, which are deposits of hyper-phosphorylated tau protein inside neurons [1]. Recent evidence has suggested that AD is not only a brain-specific disease but also a systemic disorder associated with inflammation, obesity, and other chronic diseases [2]. Specifically, substantial epidemiologic studies have reported that diabetes mellitus (DM) is a crucial risk factor for AD [3,4]. Huang et al. [3] analyzed approximately 140,000 members of the Taiwanese population and reported that newly diagnosed patients with type 2 DM (T2D) have an increased risk of AD. Furthermore, a study analyzing the Hisayama cohort suggested that DM is a significant risk factor for all-cause dementia and AD [4].

Accumulating evidence indicates that AD and DM have shared molecular mechanisms, such as oxidative stress, mitochondrial dysfunction, and inflammation [5]. Therefore, AD is referred to as “type 3 diabetes” [6]. Furthermore, several studies based on whole transcriptome analysis have identified a common pathogenesis between AD and DM. Hokama et al. [7] analyzed the postmortem brain tissues of AD patients, in Hisayama, and reported that dysregulated genes in the AD hippocampus were associated with T2D-related genes. Caberlotto et al. [8] performed microarray analyses of brain tissues from AD and DM patients, revealing several common biosignatures between the two, such as autophagy. A microarray meta-analysis of brain tissues from patients with AD and of multiple tissues (pancreas, liver, muscle, and blood) from T2D patients showed that several pathways, including the ephrin receptor, liver X receptor/retinoid X receptor, and interleukin 6, were dysregulated in both AD and T2D [9].

We previously performed a microarray meta-analysis of blood samples collected from AD patients participating in the Europe-based AddNeuroMed and US-based Alzheimer’s Disease Neuroimaging Initiative (ADNI) cohorts [10,11]. This analysis identified the immune and inflammation-, energy-, WNT-, endoplasmic reticulum-, and cancer-related pathways as the underlying mechanisms associated with the AD status [12]. Moreover, RNA-sequencing data of blood samples collected from AD Japanese subjects stored at the National Center for Geriatrics and Gerontology (NCGG) Biobank also revealed several other pathways (i.e., signal-recognition particle (SRP)-dependent cotranslational protein targeting to membrane, translational initiation, and ribosome) associated with the AD status [13].

Grayson et al. [14] performed microarray transcriptomic analyses of blood samples from T2D patients, identifying differentially expressed genes involved in the biological function of T-cell activation and signaling. Kaizer et al. [15] also reported that type 1 DM and T2D shared pathogenic mechanisms, such as overexpression of *IL1B* (probably due to hyperglycemia). Moreover, Lin et al. [16] performed RNA sequencing for neutrophils isolated from the blood of T2D patients and found that genes dysregulated in these patients were enriched in cytokine–cytokine receptor interactions, as well as nuclear factor kappa-light-chain-enhancer of activated B cells (NF-κB), tumor necrosis factor, cell adhesion molecule, Toll-like receptor, and chemokine signaling. In the OPTIMED study, whole transcriptome of T2D blood samples further revealed that genes with altered expression after anti-diabetic treatment with metformin were involved in the improvement of energy metabolism, the modulation of inflammation, and the inhibition of cancer progression [17].

Although numerous pieces of evidence suggest a shared pathophysiology between AD and DM in the brain and pancreatic tissues, few studies have compared their whole blood transcriptome signatures [18]. Santiago et al. [18] performed a microarray meta-analysis of blood RNA expression in the blood of AD and DM patients and mainly conducted differential expression (DE) analysis to identify common mechanisms between the two diseases. Another brain microarray meta-analysis study conducted a differential connectivity (DC) analysis, yielding *TYROBP* as an upstream regulator of AD [19]. The aim of our study was to identify shared blood transcriptomic signatures between AD and DM using both DE and DC analyses.

**Figure 1:**
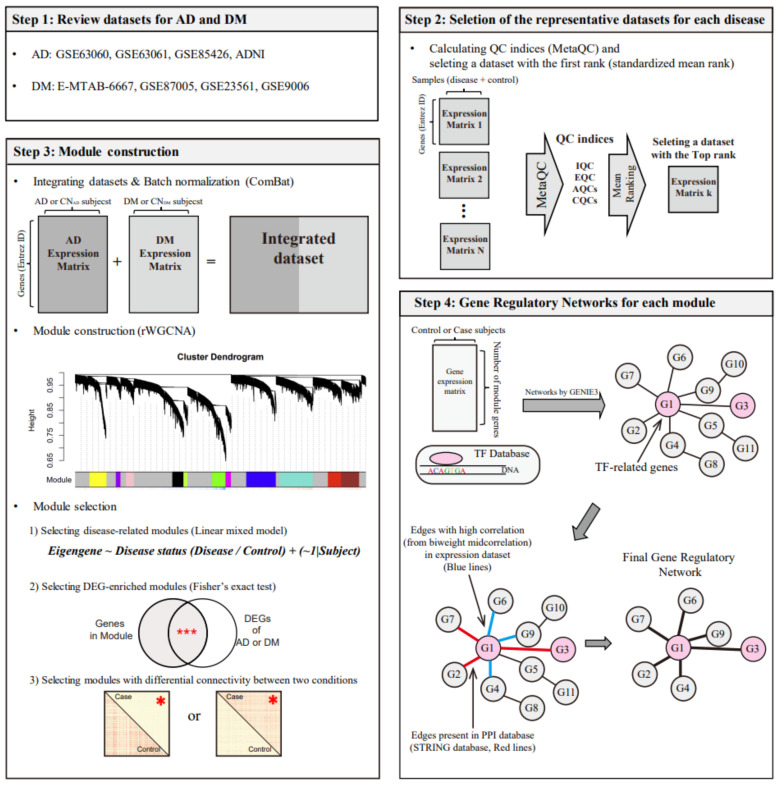
Study design for identifying common regulators between AD and DM. The symbols *** and * in step 3 indicate *p* < 0.001 (Fisher’s exact test) and *p* < 0.05 (*t*-test), respectively. AD, Alzheimer’s disease; CN_AD_, AD-matched control; DM, diabetes mellitus; CN_DM_, DM-matched control, rWGCNA, robust weighted gene co-expression network analysis; DEG, differentially expressed gene; QC, quality control; IQC, internal quality control; EQC, external quality control; AQC, accuracy quality control; CQC, consistency quality control; and TF, transcription factor.

As shown in Figure 1, we analyzed publicly available datasets for the two diseases and selected representative datasets for each disease. The two selected datasets were integrated into one large dataset, which was used for the construction of a co-expression network, yielding several modules. We selected AD- and DM-related modules based on DE and DC analyses between the disease and control groups. From the selected module, we identified common transcription factors for AD and DM using a gene regulatory network (GRN) construction method (GENIE3) [20], a transcription factor (TF) database, gene expression correlation, and a protein-protein interaction (PPI) database.

## 2. Methods

### 2.1. Datasets and Preprocessing

Initially, we collected publicly available gene expression microarray data from blood samples taken from patients with AD and DM (Table S1): Four AD datasets (ADNI, GSE63060, GSE63061, and GSE85426) and four DM datasets (GSE9006, GSE23561, GSE87005, and E-MTAB-6667). We selected appendicitis (AP) as a control disease using disease networks (Supplementary Materials) [21,22,23], and GSE9579 was selected as a transcriptomic dataset for AP. The normalization and probe selection for all datasets are described in the Supplementary Materials.

### 2.2. Selection of Representative Datasets for AD and DM

We selected representative datasets for AD and DM using a MetaQC method that provides quantitative measures for assessing the quality and consistency of microarray data for meta-analysis in the form of six indices and one summarized rank of quality for each RNA dataset [24]. The six quality control (QC) measures comprised an internal QC (IQC), an external QC (EQC), two accuracy QC indices (AQCg and AQCp), and two consistency QC indices (CQCg and CQCp). The standardized mean rank value (SMR) is the average rank of all QC indices (Supplementary Materials).

### 2.3. Comparison of Selected Blood Datasets with the Brain and Pancreas Datasets

To investigate whether the expression levels of genes in blood samples, instead of those in disease-specific tissues, can be used to show the association between diseases, we compared the expression values of all genes in a selected blood sample set with those in disease-specific tissues (brain tissues for AD and pancreas tissues for T2D). A study by Mathys et al. [25] profiled 80,660 single-cell transcriptomes of brain tissue obtained from 25 and 24 subjects with and without AD pathology, respectively. We used the summary statistics of that study (Supplementary Table S2 in the study by Mathys et al. [25]), which provided the fold changes (FCs) of the expression levels of 10,520 to 16,966 genes between AD-pathology and no-pathology tissues for five brain cell types. The pancreas dataset (GSE81608) included the whole transcriptomes of 1600 human pancreatic α, β, δ, and pancreatic polypeptide (PP) cells obtained from 6 T2D patients and 12 non-diabetic individuals [26]. We scaled the GSE81680 dataset using log-transformation.

### 2.4. Differential Expression and Pathway Analyses

We investigated differentially expressed genes (DEGs) that were common between AD and DM. DE analysis between each disease (AD or DM) and its matched control (CN_AD_ or CN_DM_) was conducted using the “lmFit” and “eBayes” functions in the limma package [27]. Results from the limma package analysis consisted of logFC values between the two conditions (disease (AD or DM) vs. healthy control (CN_AD_ or CN_DM_)) and the p-values for each gene. We defined genes with a false discovery rate (FDR)-adjusted *p*-value of less than 0.05 as DEGs. We then selected DEGs that were common between AD and DM.

We used the Dataset for Annotation, Visualization and Integrated Discovery program (DAVID) to identify enriched pathways of the DEGs [28]. We used Gene Ontology (GO) [29] and Kyoto Encyclopedia of Genes and Genomes (KEGG) [30] databases and established an FDR-adjusted p-value of less than 0.05 as the significance threshold. Using Spearman’s correlation coefficient, we compared the logFC values of all genes across AD, DM, and the control disease AP. A significance threshold for correlation across datasets was determined using a permutation test (Supplementary Materials).

### 2.5. Construction of Modules and Selection of Disease-Related Modules

First, blood RNA expression datasets for AD and DM were combined into one large dataset, after the batch effect between the AD and DM datasets was removed via the “ComBat” function in the sva package [31,32]. Subsequently, modules with the combined expression profiles were constructed using the weighted gene co-expression network analysis (WGCNA) package [33]. Specifically, a robust version of WGCNA (rWGCNA) [34,35] was used to minimize the influence of potential outlier samples. Detailed information on several hyper-parameters of the rWGCNA are described in the Supplementary Materials (Figures S1 and S2). Finally, we selected co-expression modules related to both AD and DM from the set of modules constructed with rWGCNA, using three previously established approaches. The first approach is a regression method using disease status and gene expression as independent and dependent variables, respectively [35]. The second involves selecting modules consisting of a significantly high number of DEGs [36], and the third involves selecting modules with DC between two statuses (diseased and healthy) across all pairs of genes in a module [19].

For the first approach, we used a linear mixed model (LMM), where the eigengene, which is the first principal component of the genes in a given module, and the disease status (disease or control) were used as the dependent and independent variables, respectively. We selected a module with a Bonferroni-corrected *p*-value < 0.05 for eigengene-disease associations from LMM and defined it as the disease-related module. For the second approach, Fisher’s exact test was used to evaluate whether DEGs were significantly enriched in the modules. We selected modules with a Bonferroni-corrected *p*-value < 0.05 for the number of common genes between the module genes and the DEGs from Fisher’s exact test and defined them as the DEG-enriched modules. For the third approach, the measurement of differential connectivity between the two conditions is described in the Supplementary Materials. We selected a module with a significant differential connectivity between the two conditions (Bonferroni-corrected *p*-value < 0.05) and defined it as the modular differential connectivity (MDC) module.

### 2.6. Construction of GRN and Identification of Hub Genes

For the selected module, we attempted to identify genes that were highly correlated with other genes, using the “GENIE3” function in the GENIE3 package based on the random forest algorithm with a regression tree, and constructed a GRN [20]. Two pieces of input data were needed to run GENIE3: One was an expression matrix (called exprMatrix) consisting of all candidate genes, and the other included lists of candidate upstream genes (regulators). GENIE3 generates a table with three columns: “regulatoryGene”, “targetGene”, and “weight”. The “weight” is the average of variable importance measured by the degree of variance reduction of the output variable [20]. To construct the GRN, we selected edges between two genes (regulatoryGene and targetGene) with weight values greater than the mean plus one standard deviation (SD) of the weight values.

Although GENIE3 is considered a reliable method for constructing GRNs [37], this algorithm only uses data-specific information (RNA expression) and has characteristics of randomness; therefore, it may generate biologically irrelevant regulations. To overcome these issues, we used the STRING database and correlation between two genes (Figure 1) [38]. From the edges between genes identified using GENIE3, we selected an edge when the two genes had a relationship in the STRING database or a high correlation value from the biweight midcorrelation measurement (≥95th percentile from the correlation matrices obtained from analyzing AD and DM gene expression data).

## 3. Results

### 3.1. Comparisons of Disease-Related RNA Alterations across All Blood Dataset Pairs for Each Disease

We examined whether blood expression values of different datasets from the same disease were correlated with each other. For this task, we calculated the logFC values of all genes between disease and control samples for each dataset, and then assessed their correlation between the datasets. For the four AD datasets, we measured the logFC between AD and CN_AD_ for all genes using the limma package, yielding four lists of logFC values. Then, we compared pairs of these four lists of logFC values using Spearman’s correlation. Among the four AD datasets, two AddNeuroMed datasets (GSE63060 and GSE63061) had the most highly correlated FC values (Figure S2A, Spearman’s correlation coefficient = 0.78, *p* < 0.001).

Furthermore, for the four DM datasets, we measured the logFC between DM and CN_DM_ for all genes using the limma package and then compared the logFC value of each gene for all pairs in the DM blood datasets. Three pairs exhibited positive correlations, and three pairs exhibited negative Spearman coefficient values (Figure S2B). As such, correlations among the blood datasets were not always strong, even for the same disease.

### 3.2. Selection of a Representative Dataset for Each Disease

**Figure 2:**
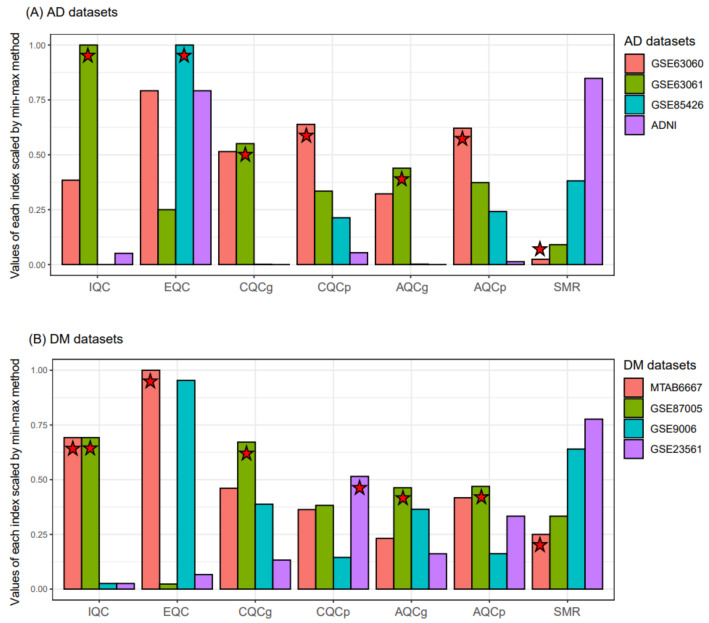
Comparison of the quality and consistency of microarray datasets using MetaQC. Six QC indices of the four AD (**A**) and four DM (**B**) datasets were measured, respectively. The QC measurements of each bar plot represents the average values. The QC indices of GSE63060 were measured three times as follows: (1) GSE63060, GSE63061, GSE85426; (2) GSE63060, GSE85426, ADNI; and (3) GSE63060, GSE85426, ADNI. The mean values of the three time measures for each QC index were calculated. Stars denote the first ranking dataset or the dataset with the best quality for each QC index. AD, Alzheimer’s disease; DM, diabetes mellitus; IQC, internal quality control; EQC, external quality control; AQCg, accuracy quality control (gene); AQCp, accuracy quality control (pathway); CQCg, consistency quality control (gene); CQCp, consistency quality control (pathway); SMR, standardized mean rank; and ADNI, Alzheimer’s Disease Neuroimaging Initiative.

Instead of using all the RNA expression datasets, as has been done in other studies [35,36], we selected representative datasets for each disease using MetaQC [24]. We measured the six QC indices from three datasets iteratively selected from the four AD datasets, resulting in an AD study having three values for each QC index. Figure 2 shows the average values of the six QC indices and the SMR scaled by min-max normalization. Based on the average values of SMR, GSE63060 and MTAB6667 had the lowest values for AD and DM, respectively. Therefore, we selected GSE63060 and MTAB667 as representative blood RNA expression datasets for AD and DM, respectively.

### 3.3. Comparison of Disease-Related RNA Alterations of the Blood with Those of the Brain and Pancreas

**Figure 3:**
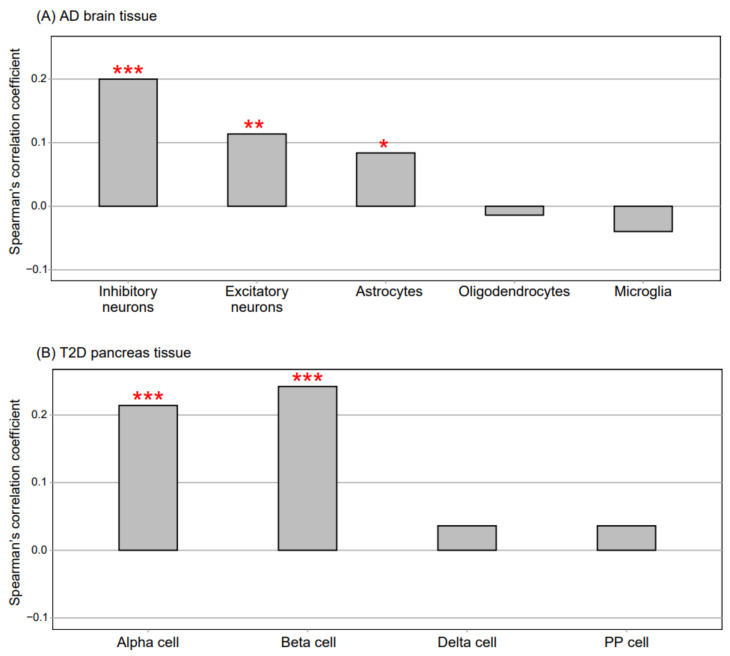
Comparison of gene expression between blood samples and disease-related tissue samples. (**A**) the AD brain dataset (single-cell transcriptomic analysis data) consisted of five cell types. Spearman’s correlation was used to compare the fold change values between AD and CN_AD_ for all genes in the AD blood dataset (GSE63060) with those of the five cell types in the AD brain dataset. (**B**) the T2D pancreas dataset (single-cell transcriptomic analysis data) consisted of four cell types. Spearman’s correlation was used to compare the fold change values between DM and CN_DM_ for all genes in the DM blood dataset (MTAB6667) with those of the four cell types in the DM pancreas dataset. ***, **, and one * indicate *p* < 0.001, *p* < 0.01, and *p* < 0.05, respectively (Spearman’s correlation). AD, Alzheimer’s disease; T2D, type 2 diabetes; PP, pancreatic polypeptide; DM, diabetes mellitus; CN_AD_, AD-matched control; and CN_DM_, DM-matched control.

We compared the FC values for all genes between each disease (AD or DM) and its respective control (CN_AD_ or CN_DM_) from the selected blood dataset with those from other disease-related tissues (the brain and pancreas) affected by AD and DM. When we compared the FC values between AD and CN_AD_ for all genes in the AD blood dataset with the FC values in the AD brain dataset comprising data for five cell types [25], the transcriptomic signatures of the AD blood data exhibited the highest consistency with those of AD inhibitory neurons, followed by the transcriptomic signatures of AD excitatory neurons and AD astrocytes (Figure 3A) (*p* < 0.05, Spearman’s correlation). When comparing the FC values between DM and CN_DM_ of the blood dataset (MTAB6667) with the FC values of the pancreas dataset comprising data for the four cell types [26], the RNA alteration patterns in the DM blood data were similar to those in the α and β cells of DM islets (*p* < 0.05, Spearman’s correlation analysis; Figure 3B). These results indicated that gene expression in the blood reflected that in the disease-related tissues.

### 3.4. Comparison of DEGs between AD and DM Blood Data

**Figure 4:**
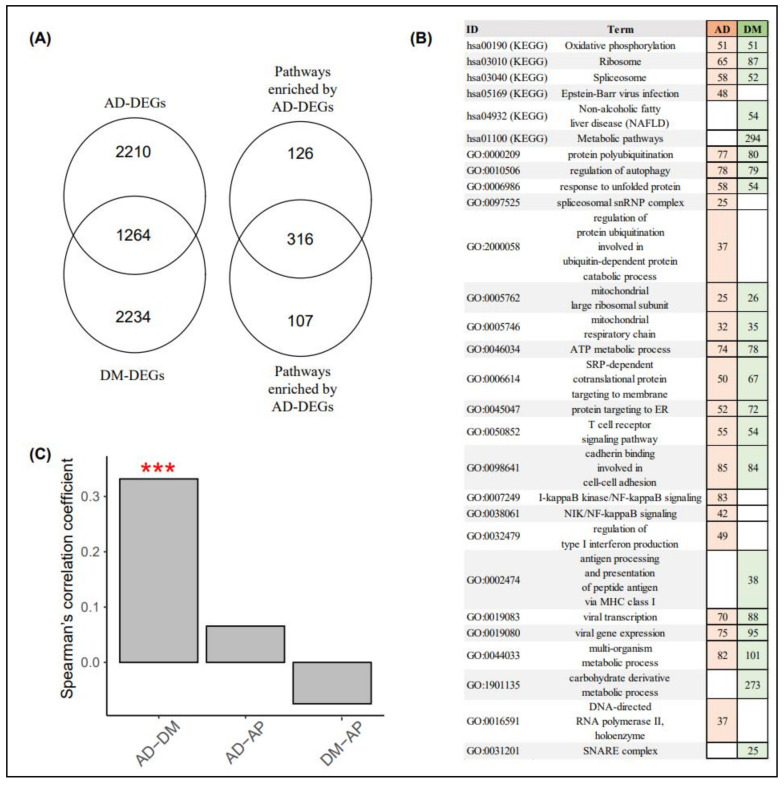
Differentially expressed genes and pathway analyses of AD and DM blood samples. (**A**) the Venn diagram on the left consists of the number of AD- and DM-DEGs, whereas the Venn diagram on the right includes the number of pathways enriched by AD- and DM-DEGs. (**B**) the reddish and greenish rectangles indicate pathways enriched via AD- and DM-DEGs, respectively. Numbers within rectangles represent the numbers of AD- and DM-DEGs in each pathway. (**C**) the *y*-axis indicates the Spearman’s correlation coefficient in the FC values for all genes between the disease and control samples in the AD, DM, and AP blood datasets. (***, *p* < 0.001; permutation test). AD, Alzheimer’s disease; DM, diabetes mellitus; AP, appendicitis; AD-DEG, differentially expressed genes between AD and AD-matched controls; DM-DEG, differentially expressed genes between DM and DM-matched controls; and FC, fold change.

The blood datasets for AD and DM consisted of 11,342 and 16,592 genes, respectively, and the number of genes shared between the two datasets was 10,172. We identified 3474 and 3498 DEGs between AD and CN_AD_ and DM and CN_DM_, respectively (Figure 4A). The DEGs between AD and CN_AD_ (AD-DEGs) significantly overlapped with the DEGs between DM and CN_DM_ (DM-DEGs). The number of DEGs shared between the AD-DEGs and DM-DEGs was 1264 (*p* = 0.002, hypergeometric test). In the pathway analyses, 442 and 423 pathways were enriched by AD- and DM-DEGs, respectively (Figure 4A). The pathways enriched by the AD-DEGs also significantly overlapped with those enriched by the DM-DEGs. The number of shared pathways was 316 (*p* < 5.92 × 10^−310^). Figure 4B shows notable pathways enriched by AD- and DM-DEGs.

The logFC values for all genes between AD and CN_AD_ were significantly correlated with those for all genes between DM and CN_DM_ (Figure 4C; Spearman’s correlation coefficient = 0.332, *p* < 0.001, permutation test). In contrast, the logFC values of the AD and DM blood datasets were not significantly related to those of the AP blood dataset (the control).

### 3.5. Identification of the AD and DM Co-Related Module

**Figure 5:**
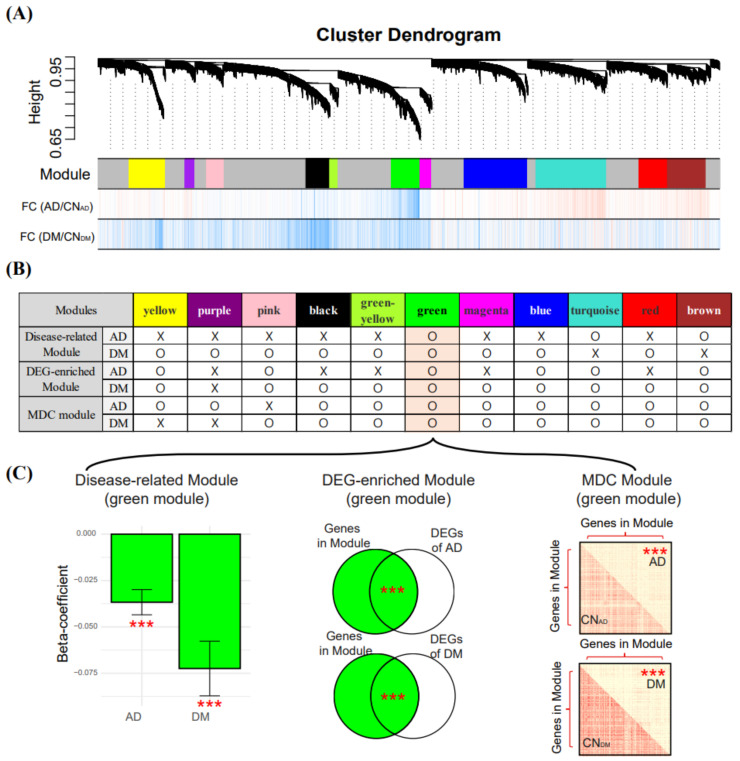
Module construction using rWGCNA and selection of AD and DM co-related modules. (**A**) modules described by different colors were constructed using rWGCNA. FC values (logFC) between two conditions were determined using the limma package. Vertical bars with red and blue colors indicate genes with logFC > 0 and logFC < 0, respectively. (**B**) three criteria (described in the Section 2) were used to select modules. The “O” indicates that a module has a Bonferroni-corrected *p*-value < 0.05 for each criterion and denotes modules suitable for selection, and the “X” indicates that a module does not satisfy these criteria. (**C**) in the bar plot on the left, the *y*-axis indicates the beta-coefficient obtained from LMM, with eigengene and disease status as the dependent and independent variables, respectively. *** indicate the *p*-value (***, Bonferroni-corrected *p*-value < 0.001) from the LMM. In the middle Venn diagrams, the *p*-values between genes in the module and the DEGs were measured using Fisher’s exact test. In the matrix plots on the right, the red and yellow colors in squares indicate high or low biweight midcorrelation values, respectively. *** indicate a significant difference (*t*-test) between the intra-modular connectivity of the control and the case. rWGCNA, robust weighted gene co-expression network analysis; AD, Alzheimer’s disease; DM, diabetes mellitus; CN_AD_, AD-matched control; CN_DM_, DM-matched control; DEG, differentially expressed gene; and LMM, linear mixed model.

By applying rWGCNA to the integrated blood dataset, we constructed 11 modules consisting of genes with similar expression patterns across samples (Figure 5A). We examined these 11 modules using the three approaches described in the Section 2 and selected regression-based disease-related modules, DEG-enriched modules, and MDC modules (Figure 5B). For AD, we selected green, turquoise, and brown modules that were related to AD (had a significant association with AD status and module-eigengene), significantly overlapped with the AD-DEGs, and demonstrated a significant differential connectivity between AD and CN_AD_. For DM, we selected seven modules (green, pink, black, greenyellow, magenta, blue, and red) that were shown via the three approaches to be related to DM. The green module was selected for both AD and DM; therefore, we defined the green module as the co-related module between AD and DM (Figure 5C).

The green module had 467 genes, of which 452 and 306 genes were AD- and DM-DEGs, respectively (middle Venn diagram in Figure 5C). The eigengene of the 467 genes in the green module had a negative relationship with the presence of AD or DM (left bar plot in Figure 5C), indicating that most genes in the green module had higher expressions in AD and DM than in CN_AD_ and CN_DM_. The 467 genes in the green module had lower connectivity in AD and DM (according to the biweight midcorrelation) than in CN_AD_ and CN_DM_ (right matrix plots in Figure 5C).

The 467 genes in the green module were enriched in 252 KEGG and GO pathways (Table S2), and the representative pathways included Alzheimer’s disease (hsa05010), ribosome (hsa03010), oxidative phosphorylation (hsa00190), mitochondrial respiratory chain (GO:0005746), and protein targeting to endoplasmic reticulum (ER) (GO:0045047). These pathways are known to be related to AD and DM [39,40,41,42,43,44].

### 3.6. GRN and Identification of Hub Genes

Using GENIE3 [20], we constructed a GRN for the green module, which was related to both AD and DM. The green module contained 42 of the 2764 known TF-related genes [45]. Four GRNs for AD, CN_AD_, DM, and CN_DM_ were constructed using 467 genes in the green module and 42 TF-related genes as candidate genes and regulators (input features of GENIE3), respectively. Among the edges obtained using GENEI3, we only selected edges when an edge had PPI evidence in the STRING database or a high biweight midcorrelation value (≥95th percentile).

**Figure 6:**
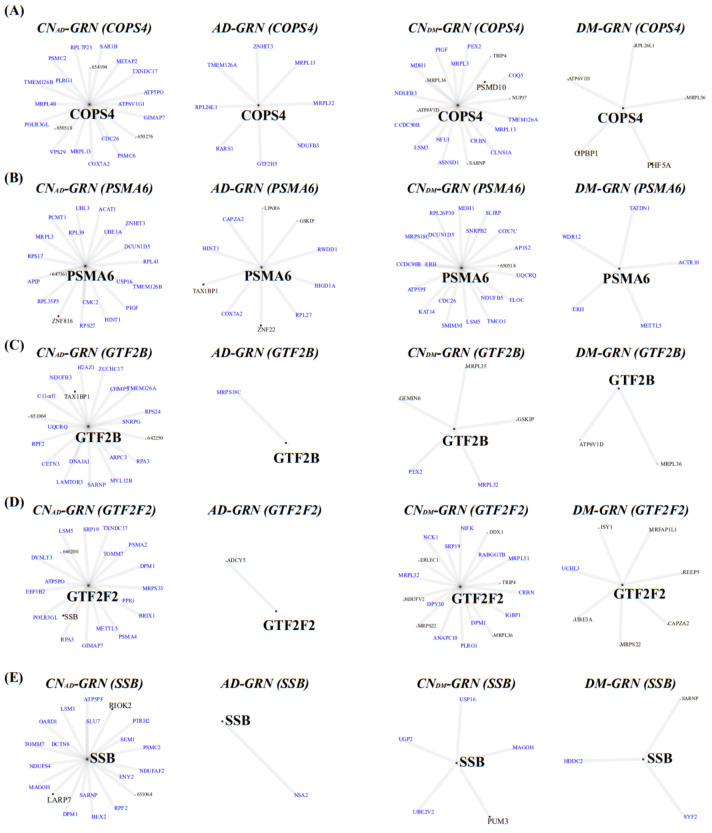
Five shared regulatory genes in GRNs. Four GRNs for (**A**) *COPS4*, (**B**) *PSMA6*, (**C**) *GTF2B*, (**D**) *GTF2F2*, and (**E**) *SSB* were established using the AD, CN_AD_, DM, and CN_DM_ blood samples, respectively. GRNs were constructed by integrating GENIE3, the TF database, the PPI database, and correlation values of gene expression. Blue colors indicate down-regulated genes. A large size and black color indicate TF-related genes. Edges between two genes were constructed from GENIE3, the PPI database, and correlations of RNA expression data. Four GRNs per gene, constructed using AD, CN_AD_, DM, and CN_DM_ samples, respectively, are illustrated. AD, Alzheimer’s disease; DM, diabetes mellitus; CN_AD_, AD-matched control; CN_DM_, DM-matched control; GRN, gene regulatory network; TF, transcription factor; and PPI, protein-protein interaction.

Among the 42 TF-related genes, 24 AD-GRN genes had fewer edges than the CN_AD_-GRN genes, while 18 AD-GRN genes had greater than or equal the number of edges of the CN_AD_-GRN genes. Among the 42 TF-related genes, 11 DM-GRN genes had fewer edges than the CN_DM_-GRN genes, while 31 DM-GRN genes had greater than or equal the number of edges of the CN_DM_-GRN genes. Because the genes in the green module of the AD- and DM-GRNs generally had lower connectivity or correlational values than those of the matched CN-GRNs (Figure 5), we focused on TF-related genes with fewer edges in the disease-GRN than in the CN-GRN. The following five genes were shared between the 24 and 11 genes with fewer edges in the AD- and DM-GRNs, respectively, than in the matched CN-GRNs: *COPS4*, *PSMA6*, *GTF2B*, *GTF2F2*, and *SSB* (Figure 6). This finding suggested that these five genes were dysregulated TFs shared by AD and DM.

### 3.7. Validation of the Green Module and the Five Hub Genes

**Figure 7:**
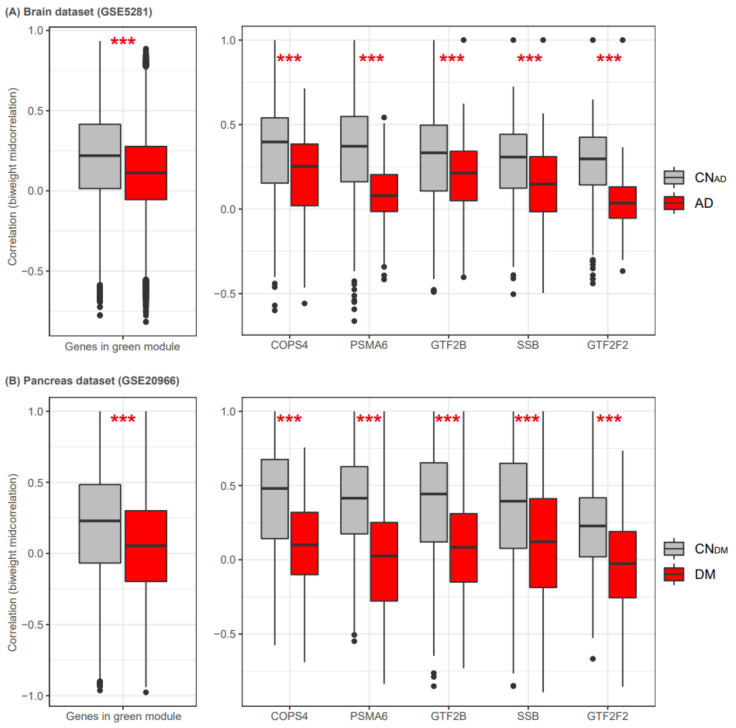
Five shared regulatory genes in brain and pancreas datasets. We measured the differential connectivity of the green module in datasets for AD and CN_AD_ brain tissue samples (**A**) and DM and CN_DM_ pancreas tissue samples (**B**). The boxplots on the left indicate biweight midcorrelation values from all possible pairs of genes in the green module. The boxplots on the right indicate the correlation values (biweight midcorrelation) of each gene with other genes in the green module. Gray and red boxplots show the correlation values for CN_AD_/CN_DM_ and AD/DM, respectively. AD, Alzheimer’s disease; DM, diabetes mellitus; CN_AD_, AD-matched control; and CN_DM_, DM-matched control. ***, *p* < 0.001 (*t*-test).

To compare the differential connectivity of the green module between the disease and control blood samples with that of other tissues, we analyzed gene expression in datasets for AD and CN_AD_ brain tissue samples (GSE5281) and DM and CN_DM_ pancreas tissue samples (GSE20966). Detailed information on the pre-processing of the two datasets is presented in the Supplementary Materials. The brain tissue dataset (GSE5281) contained gene expression values for 420 of the 467 genes in the green module. Using all pairs of the 420 genes, we constructed two separate biweight midcorrelation matrices for AD and CN_AD_ samples. The results showed that the 420 genes in the AD brain had lower correlation values than those in the CN_AD_ brain (*p* < 2.2 × 10^−16^, *t*-test). Specifically, the five genes *COPS4*, *PSMA6*, *GTF2B*, *GTF2F2*, and *SSB* had lower correlation values in the AD dataset than in the CN_AD_ dataset (Figure 7). The pancreas dataset (GSE20966) contained gene expression values for 435 of the 467 green module genes. We also constructed two separate biweight midcorrelation matrices for DM and CN_DM_ using all pairs of the 435 genes. The 435 genes had lower correlational values in the DM pancreas dataset than in the CN_DM_ pancreas dataset (*p* < 2.2 × 10^−16^, *t*-test). Additionally, the five TF-related genes had lower correlation values in the DM dataset than in the CN_DM_ dataset. These results confirmed that these five genes had differential connectivity in disease-related tissues.

## 4. Discussion

In this study, we identified five TF-related genes (*COPS4*, *PSMA6*, *GTF2B*, *GTF2F2*, and *SSB*) in the blood that were dysregulated in both AD and DM. These results were consistent in disease-related tissues (the brain for AD and the pancreas for DM).

*COPS4* is a component of the *COP9* signalosome and removes the ubiquitin-like protein Nedd8, mediating the reduction of the misfolded protein burden, which is the crucial mechanism of AD [46]. Additionally, the *COP9* signalosome is related to obesity, which is the major risk factor for T2D, by mediating the expression of the *C/EBP* homologous protein and regulating the differentiation of pre-adipocytes. Similarly, *PSMA6* is a component of the 20S proteasome and is related to both AD and DM [47,48]. Several studies have reported that *PSMA6* has a possible role in DM complications, such as myocardial infarction and nephropathy [49,50].

A text-mining study that predicted physical interactions among candidate disease genes and constructed a molecular network analysis reported *GTF2B* as the top AD-related gene [51]. Nilsson et al. [52] analyzing transcriptomic data from adipose tissues in a twin cohort, reported *GTF2B* as a down-regulated TF-related gene in the T2D group. *GTF2F2*, which exists in many tissues and organs, plays a role in both the initiation and elongation of transcription by controlling RNA polymerase II [53]. Recently, *GTF2F2* was reported to be involved in neurogenesis, neuroplasticity, and synaptogenesis by mediating *NRF1* [54].

Numerous studies have focused on the autoantibody for *SSB* (anti-*SSB*), whose presence characterizes the main pathophysiology of Sjogren’s syndrome and is not part of the normal function of *SSB*. *SSB* is known as a transcription termination factor that facilitates the termination of transcription by RNA polymerase III [55]. Xu et al. [36] performed a meta-microarray analysis of brain tissue and reported that the *SSB* gene was dysregulated in AD patients. A separate study analyzing blood gene expression reported that *SSB* was also dysregulated in AD samples [56].

In this study, we selected a representative gene expression dataset for each disease instead of integrating several gene expression datasets, for the following three reasons: First, the disease-related gene expression values were variable, even across samples of the same disease. Among all datasets in each disease, only one pair of the AD datasets (GSE63060/GSE63061) and two pairs of the DM blood datasets had SCC > 0.15 when comparing their FC values for all genes (Figure S3). Second, when we compared the logFC changes between AD and CN_AD_ in the large AD blood dataset with the logFC changes between DM and CN_DM_ in the large DM blood dataset, the correlation between AD and DM was insignificant (Figure S4). Third, too many genes were excluded when generating a large dataset given that the platforms used (e.g., microarray) had different coverage for transcripts or genes. For example, we integrated seven different microarray datasets that resulted in only about 1000 genes found to be present in all datasets (Figure S4).

In this study, we identified the shared transcriptomic signatures between AD and DM by integrating co-expression, DE, and DC analyses. Numerous studies used the DE method between two statuses (disease and control) to identify genes related with a specific disease [7,8,9,12,13,14]. However, the DE-based methods cannot reveal two genes that are simultaneously upregulated or downregulated. Therefore, alternative analyses, such as co-expression and DC analyses, have been used to uncover coordinated and regulatory biological signatures [19,35]. This combination of DE and DC analyses allowed us identify a shared module between AD and DM containing highly correlated AD- and DM-related genes.

The selection of upstream genes, a crucial task in numerous studies, has been performed using various methods. Zhang et al. [19] selected the upstream or parent genes by integrating multi-omics datasets. Briefly, they implemented a Bayesian approach by allowing genes with cis-expression quantitative trait loci as the upstream or parent genes [19]. In turn, the Stockholm Atherosclerosis Gene Expression study used genes in TF-related GO terms as the upstream or hub genes [57]. Herein, we used 2765 TF-related genes, which were manually curated from previously validated studies and several databases [45], as well as the PPI database [38] to reduce false positive findings.

Lastly, we selected the five genes (*COPS4*, *PSMA6*, *GTF2B*, *GTF2F2*, and *SSB*) by strict criteria (e.g., statistically significance in DC and DE analyses, the presence of interaction with other genes in the PPI network, and the high correlation with other genes). However, there were no previous in vitro or in vivo studies reporting associations between the five genes and AD or DM. This could be due to the small number of previous studies that have investigated shared transcriptome changes between AD and DM. However, because our study was performed based on integration of multiple datasets and unbiased analyses, we expect that the identified genes are biomarkers of AD and DM.

## 5. Conclusions

In summary, our study revealed a gene module in which genes were commonly dysregulated in both AD and DM blood samples. These genes belonged to AD- and DM-related pathways.

## Data Availability

Gene expression datasets are publicly available (ADNI, http://adni.loni.usc.edu/; GEO, https://www.ncbi.nlm.nih.gov/geo/; ArrayExpress, https://www.ebi.ac.uk/arrayexpress/). A complete listing of ADNI investigators can be found at: http://adni.loni.usc.edu/wp-content/uploads/how_to_apply/ADNI_Acknowledgement_List.pdf.

## Supplementary Materials

The following are available online at https://www.mdpi.com/2227-9059/9/1/34/s1, Figure S1: Soft Threshold Power (Hyper-parameter) of Co-Expression Network (rWGCNA), Figure S2: DS, MMS, DCOR, PAM (Hyper-parameters) of Co-Expression Network (rWGCNA), Figure S3: Correlation matrices from Spearman correlation for all pairwise comparisons among blood datasets in each disease, Figure S4: Comparison of gene expressions between integrated AD blood and integrated DM blood datasets, Table S1: All analyzed datasets (AD and DM), Table S2: Enriched pathways of genes in green module.

## References

[1] Kumar A., Singh A. (2015). A review on Alzheimer’s disease pathophysiology and its management: An update. Pharmacol. Rep..

[2] Morris J.K., Honea R.A., Vidoni E.D., Swerdlow R.H., Burns J. (2014). Is Alzheimer’s disease a systemic disease?. Biochim. Biophys. Acta Mol. Basis Dis..

[3] Huang C.-C., Chung C.-M., Leu H.-B., Lin L.-Y., Chiu C.-C., Hsu C.-Y., Chiang C.-H., Huang P.-H., Chen T.-J., Lin S.-J. (2014). Diabetes Mellitus and the Risk of Alzheimer’s Disease: A Nationwide Population-Based Study. PLoS ONE.

[4] Ohara T., Doi Y., Ninomiya T., Hirakawa Y., Hata J., Iwaki T., Kanba S., Kiyohara Y. (2011). Glucose tolerance status and risk of dementia in the community: The Hisayama Study. Neurology.

[5] Kandimalla R., Thirumala V., Reddy P.H. (2017). Is Alzheimer’s disease a Type 3 Diabetes? A critical appraisal. Biochim. Biophys. Acta Mol. Basis Dis..

[6] De La Monte S.M., Wands J.R. (2008). Alzheimer’s Disease is Type 3 Diabetes—Evidence Reviewed. J. Diabetes Sci. Technol..

[7] Hokama M., Oka S., Leon J., Ninomiya T., Honda H., Sasaki K., Iwaki T., Ohara T., Sasaki T., LaFerla F.M. (2014). Altered Expression of Diabetes-Related Genes in Alzheimer’s Disease Brains: The Hisayama Study. Cereb. Cortex.

[8] Caberlotto L., Nguyen T.-P., Lauria M., Priami C., Rimondini R., Maioli S., Cedazo-Minguez A., Sita G., Morroni F., Corsi M. (2019). Cross-disease analysis of Alzheimer’s disease and type-2 Diabetes highlights the role of autophagy in the pathophysiology of two highly comorbid diseases. Sci. Rep..

[9] Mirza Z., Kamal M.A., Buzenadah A.M., Al-Qahtani M.H., Karim S. (2014). Establishing Genomic/Transcriptomic Links Between Alzheimer’s Disease and Type 2 Diabetes Mellitus by Meta-Analysis Approach. CNS Neurol. Disord. Drug Targets.

[10] Lovestone S., Francis P., Kloszewska I., Mecocci P., Simmons A., Soininen H., Spenger C., Tsolaki M., Vellas B., Wahlund L.O. (2009). AddNeuroMed—the European collaboration for the discovery of novel biomarkers for Alzheimer’s disease. Ann. N. Y. Acad. Sci..

[11] Mueller S.G., Weiner M., Thal L.J., Petersen R.C., Jack C.R., Jagust W., Trojanowski J.Q., Toga A.W., Beckett L. (2005). Ways toward an early diagnosis in Alzheimer’s disease: The Alzheimer’s Disease Neuroimaging Initiative (ADNI). Alzheimer Dement..

[12] Lee T., Lee H. (2020). Prediction of Alzheimer’s disease using blood gene expression data. Sci. Rep..

[13] Shigemizu D., Mori T., Akiyama S., Higaki S., Watanabe H., Sakurai T., Niida S., Ozaki K. (2020). Identification of potential blood biomarkers for early diagnosis of Alzheimer’s disease through RNA sequencing analysis. Alzheimer Res. Ther..

[14] Grayson B.L., Wang L., Aune T.M. (2011). Peripheral blood gene expression profiles in metabolic syndrome, coronary artery disease and type 2 diabetes. Genes Immun..

[15] Kaizer E.C., Glaser C.L., Chaussabel D., Banchereau J., Pascual V., White P.C. (2007). Gene Expression in Peripheral Blood Mononuclear Cells from Children with Diabetes. J. Clin. Endocrinol. Metab..

[16] Lin Q., Zhou W., Wang Y., Huang J., Hui X., Zhou Z., Xiao Y. (2020). Abnormal Peripheral Neutrophil Transcriptome in Newly Diagnosed Type 2 Diabetes Patients. J. Diabetes Res..

[17] Ustinova M., Ansone L., Silamikelis I., Rovite V., Elbere I., Silamikele L., Kalnina I., Fridmanis D., Sokolovska J., Konrade I. (2020). Whole-blood transcriptome profiling reveals signatures of metformin and its therapeutic response. PLoS ONE.

[18] Santiago J.A., Bottero V., Potashkin J.A. (2019). Transcriptomic and Network Analysis Highlight the Association of Diabetes at Different Stages of Alzheimer’s Disease. Front. Neurosci..

[19] Zhang B., Gaiteri C., Bodea L.-G., Wang Z., McElwee J., Podtelezhnikov A.A., Zhang C., Xie T., Tran L., Dobrin R. (2013). Integrated Systems Approach Identifies Genetic Nodes and Networks in Late-Onset Alzheimer’s Disease. Cell.

[20] Huynh-Thu V.A., Irrthum A., Wehenkel L., Geurts P. (2010). Inferring Regulatory Networks from Expression Data Using Tree-Based Methods. PLoS ONE.

[21] Zhou X., Menche J., Barabási A.-L., Sharma A. (2014). Human symptoms-disease network. Nat. Commun..

[22] Menche J., Sharma A., Kitsak M., Ghiassian S.D., Vidal M., Loscalzo J., Barabási A.-L. (2015). Uncovering disease-disease relationships through the incomplete interactome. Science.

[23] Kim J., Kim J.-J., Lee H. (2017). An analysis of disease-gene relationship from Medline abstracts by DigSee. Sci. Rep..

[24] Kang D.D., Sibille E., Kaminski N., Tseng G. (2011). MetaQC: Objective quality control and inclusion/exclusion criteria for genomic meta-analysis. Nucleic Acids Res..

[25] Mathys H., Davila-Velderrain J., Peng Z., Gao F., Mohammadi S., Young J.Z., Menon M., He L., Abdurrob F., Jiang X. (2019). Single-cell transcriptomic analysis of Alzheimer’s disease. Nat. Cell Biol..

[26] Xin Y., Kim J., Okamoto H., Ni M., Wei Y., Adler C., Murphy A.J., Yancopoulos G.D., Lin C., Gromada J. (2016). RNA Sequencing of Single Human Islet Cells Reveals Type 2 Diabetes Genes. Cell Metab..

[27] Smyth G.K. (2005). Limma: Linear Models for Microarray Data. Bioinformatics and Computational Biology Solutions Using R and Bioconductor.

[28] Huang D.W., Sherman B.T., Lempicki R.A. (2009). Systematic and integrative analysis of large gene lists using DAVID bioinformatics resources. Nat. Protoc..

[29] (2019). The Gene Ontology Consortium The Gene Ontology Resource: 20 years and still GOing strong. Nucleic Acids Res..

[30] Kanehisa M., Goto S. (2000). KEGG: Kyoto Encyclopedia of Genes and Genomes. Nucleic Acids Res..

[31] Leek J.T., Johnson W.E., Parker H.S., Jaffe A.E., Storey J.D. (2012). The sva package for removing batch effects and other unwanted variation in high-throughput experiments. Bioinformatics.

[32] Johnson W.E., Li C., Rabinovic A. (2006). Adjusting batch effects in microarray expression data using empirical Bayes methods. Biostatistics.

[33] Langfelder P., Horvath S. (2008). WGCNA: An R package for weighted correlation network analysis. BMC Bioinform..

[34] Parikshak N.N., Luo R., Zhang A., Won H., Lowe J.K., Chandran V., Horvath S., Geschwind D.H. (2013). Integrative Functional Genomic Analyses Implicate Specific Molecular Pathways and Circuits in Autism. Cell.

[35] Gandal M.J., Haney J.R., Parikshak N.N., Leppa V., Ramaswami G., Hartl C., Schork A.J., Appadurai V., Buil A., Werge T. (2018). Shared molecular neuropathology across major psychiatric disorders parallels polygenic overlap. Science.

[36] Xu M., Zhang D.-F., Luo R., Wu Y., Zhou H., Kong L.-L., Bi R., Yao Y.-G. (2018). A systematic integrated analysis of brain expression profiles reveals YAP1 and other prioritized hub genes as important upstream regulators in Alzheimer’s disease. Alzheimers Dement..

[37] Pratapa A., Jalihal A.P., Law J.N., Bharadwaj A., Murali T.M. (2020). Benchmarking algorithms for gene regulatory network inference from single-cell transcriptomic data. Nat. Methods.

[38] Szklarczyk D., Gable A.L., Lyon D., Junge A., Wyder S., Huerta-Cepas J., Simonovic M., Doncheva N.T., Morris J.H., Bork P. (2019). STRING v11: Protein–protein association networks with increased coverage, supporting functional discovery in genome-wide experimental datasets. Nucleic Acids Res..

[39] Ding Q., Markesbery W.R., Chen Q., Li F., Keller J.N. (2005). Ribosome Dysfunction Is an Early Event in Alzheimer’s Disease. J. Neurosci..

[40] Ashford A.J., Pain V.M. (1986). Effect of diabetes on the rates of synthesis and degradation of ribosomes in rat muscle and liver in vivo. J. Biol. Chem..

[41] Liang W.S., Reiman E.M., Valla J., Dunckley T., Beach T.G., Grover A., Niedzielko T.L., Schneider L.E., Mastroeni D., Caselli R. (2008). Alzheimer’s disease is associated with reduced expression of energy metabolism genes in posterior cingulate neurons. Proc. Natl. Acad. Sci. USA.

[42] Sivitz W.I., Yorek M.A. (2010). Mitochondrial Dysfunction in Diabetes: From Molecular Mechanisms to Functional Significance and Therapeutic Opportunities. Antioxid. Redox Signal..

[43] Chadwick W., Mitchell N., Martin B., Maudsley S. (2012). Therapeutic targeting of the endoplasmic reticulum in Alzheimer’s disease. Curr. Alzheimer Res..

[44] Dong Y., Fernandes C., Liu Y., Wu Y., Wu H., Brophy M.L., Deng L., Song K., Wen A., Wong S. (2017). Role of endoplasmic reticulum stress signalling in diabetic endothelial dysfunction and atherosclerosis. Diabetes Vasc. Dis. Res..

[45] Lambert S.A., Jolma A., Campitelli L.F., Das P.K., Yin Y., Albu M., Chen X., Taipale J., Hughes T.R., Weirauch M.T. (2018). The Human Transcription Factors. Cell.

[46] Parry T.L., Melehani J.H., Ranek M.J., Willis M.S. (2015). Functional Amyloid Signaling via the Inflammasome, Necrosome, and Signalosome: New Therapeutic Targets in Heart Failure. Front. Cardiovasc. Med..

[47] Upadhya S.C., Hegde A.N. (2007). Role of the ubiquitin proteasome system in Alzheimer’s disease. BMC Biochem..

[48] Broca C., Varin E., Armanet M., Tourrel-Cuzin C., Bosco M., Dalle S., Wojtusciszyn A. (2014). Proteasome Dysfunction Mediates High Glucose-Induced Apoptosis in Rodent Beta Cells and Human Islets. PLoS ONE.

[49] Barbieri M., Marfella R., Rizzo M.R., Boccardi V., Siniscalchi M., Schiattarella C., Siciliano S., Lemme P., Paolisso G. (2008). The—8 UTR C/G polymorphism of PSMA6 gene is associated with susceptibility to myocardial infarction in type 2 diabetic patients. Atherosclerosis.

[50] Feng Y., Jin M.-Y., Liu D.-W., Dong-Wei L. (2018). Proteasome subunit-α type-6 protein is post-transcriptionally repressed by the microRNA-4490 in diabetic nephropathy. Biosci. Rep..

[51] Krauthammer M., Kaufmann C.A., Gilliam T.C., Rzhetsky A. (2004). Molecular triangulation: Bridging linkage and molecular-network information for identifying candidate genes in Alzheimer’s disease. Proc. Natl. Acad. Sci. USA.

[52] Nilsson E.A., Jansson P.A., Perfilyev A., Volkov P., Pedersen M., Svensson M.K., Poulsen P., Ribel-Madsen R., Pedersen N.L., Almgren P. (2014). Altered DNA Methylation and Differential Expression of Genes Influencing Metabolism and Inflammation in Adipose Tissue From Subjects With Type 2 Diabetes. Diabetes.

[53] Tan S., Aso T., Conaway R.C., Conaway J.W. (1994). Roles for both the RAP30 and RAP74 subunits of transcription factor IIF in transcription initiation and elongation by RNA polymerase II. J. Biol. Chem..

[54] Preciados M., Yoo C., Roy D. (2016). Estrogenic Endocrine Disrupting Chemicals Influencing NRF1 Regulated Gene Networks in the Development of Complex Human Brain Diseases. Int. J. Mol. Sci..

[55] Gottlieb E., Steitz J.A. (1989). Function of the mammalian La protein: Evidence for its action in transcription termination by RNA polymerase III. EMBO J..

[56] Naughton B.J., Duncan F.J., Murrey D.A., Meadows A.S., Newsom D.E., Stoicea N., White P., Scharre D.W., Mccarty D.M., Fu H. (2014). Blood Genome-Wide Transcriptional Profiles Reflect Broad Molecular Impairments and Strong Blood-Brain Links in Alzheimer’s Disease. J. Alzheimer Dis..

[57] Hägg S., Skogsberg J., Lundström J., Noori P., Nilsson R., Zhong H., Maleki S., Shang M.-M., Brinne B., Bradshaw M. (2009). Multi-Organ Expression Profiling Uncovers a Gene Module in Coronary Artery Disease Involving Transendothelial Migration of Leukocytes and LIM Domain Binding 2: The Stockholm Atherosclerosis Gene Expression (STAGE) Study. PLoS Genet..

