# Supplementary material for: Shared Blood Transcriptomic Signatures between Alzheimer’s Disease and Diabetes Mellitus †
Source: Biomedicines. 2021 Jan 4;9(1):34. doi: 10.3390/biomedicines9010034 (PMC7823888; doi:10.3390/biomedicines9010034)
Supplement: Supplementary file 1 [file biomedicines-09-00034-s001.pdf]

# Shared Blood Transcriptomic Signatures between Alzheimer's Disease and Diabetes Mellitus

(Supplementary Material)

Taesic Lee[1] and Hyunju Lee[1, 2, 3]

[1]Department of Biomedical Science and Engineering, Gwangju Institute of Science and Technology, Gwangju, South Korea.

[2]Artificial Intelligence Graduate School, Gwangju Institute of Science and Technology, Gwangju, South Korea.

[3]School of Electrical Engineering and Computer Science, Gwangju Institute of Science and Technology, Gwangju, South Korea.

**Corresponding author**

Hyunju Lee, PhD

Professor, School of Electrical Engineering and Computer Science

Artificial Intelligence Graduate School

Gwangju institute of science and technology

## Selection of Appendicitis as the control disease (For 2.1 section in Manuscript)

To select control disease irrelevant with AD, we used the two disease-disease networks, including the human symptom disease network (HSDN) [1] and interactome-separation network (ISN) [2, 3]. HSDN estimated relationship between two diseases via cosine similarity of Term Frequency-Inverse Document Frequency of symptom values that were previously curated from PubMed [1]. For ISN, we used disease-related genes curated from DigSee [3] and PPI network [2], constructed disease modules, and calculated the degree of relationship between two diseases via modular distance. As a result, 146 diseases [i.e., cholangitis, diverticulitis, and appendicitis (AP)] were resulted to be irrelevant with AD in both HSDN and ISN.

## Preprocessing of blood RNA datasets (For 2.1 section in Manuscript)

We downloaded ADNI expression data normalized by Robust Multi-Array Analysis (RMA) method [4]. We downloaded normalized datasets (Accession ID: GSE63060 and GSE63061) from the Gene Expression Omnibus database (GEO) [5]. We downloaded "GSE85426 raw data.txt" from the GEO, then scaled and normalized GSE85426 via exponential function and quantile normalization (normalizeQuantiles function in limma package), respectively.

E-MTAB-6667 was normalized by background correction and quantile normalization via 'neqc' function in lumi package. We used RNA expression data (E-MTAB-6667) of CD14 positive blood samples. Because E-MTAB-6667 included the unbalanced number of samples (19 diabetes mellitus (DM) patients and 291 healthy controls), we resampled 40 subjects among 291 healthy controls without replacement. We iterated the resampling at 100 times, and in each iteration, we measured Spearman's correlation coefficient value between log[fold change (FC)] values for all genes between two conditions of the original dataset and those of resampled dataset. The logFC values were RNA expression alteration between control and DM, measured by Limma method [6]. The number of logFC values were same with the number of genes in RNA expression set (i.e., if a RNA expression data consists of N number of genes, we can get N number of FC values). We selected a resampled dataset with the highest correlation value (Spearman correlation coefficient = 0.9894).

We downloaded GSE87005 pre-processed by the locally weighted scatterplot smoothing (LOWESS) algorithm [7], and normalized the downloaded dataset via quantile normalization (normalizeQuantiles function in limma package). GSE23561 was normalized by quantile normalization (normalizeQuantiles function in limma package). We scaled and normalized GSE9006 via log transformation and quantile normalization (normalizeQuantiles function in limma package), respectively.

We scaled and normalized GSE9579 (# of controls = 4, # of AP = 9) via log transformation and quantile normalization (normalizeQuantiles function in limma package), respectively.

For all RNA expression datasets, we excluded approximately 30% of the probes that had small standard deviations across samples, then about 30% of the under-expressed probes based on the small mean expression values across subjects. To systemically compare gene expression of different platforms, we mapped all probes or probe-sets ID to Entrez ID, and we eliminated probes without matched Entrez IDs. For multiple probes annotated with a gene (Entrez ID), we selected the probe with the maximum average expression value via 'collapseRows' function in Weighted Gene Co-Expression Network Analysis (WGCNA) package[8].

## MetaQC (For 2.2 section in Manuscript)

Using the 'MetaQC' and 'runQC' functions in the MetaQC package, we calculated the levels of the six QC indices and SMR for each RNA expression dataset. Because pathway information was needed to measure the six QC indices in MetaQC, we downloaded the Kyoto Encyclopedia of Genes and Genomes (KEGG)[9] database from MSigDB[10] To run the MetaQC algorithm, at least three RNA datasets were needed. If we had used the MetaQC method to analyze four RNA datasets for each disease, only 1000 to 2000 genes would have been available for measuring QC, because only genes that were present across all studies were analyzed. Therefore, each time, we measured the QC of three RNA datasets that were the minimum size required to run the MetaQC. As a result, four combinations $[_{4(\text{four AD or DM RNA datasets})}C_{3(\text{minimum number of sets for MetaQC})}]$ of the MetaQC were calculated.

## Permutation test (For 2.4 section in Manuscript)

For each study (AD, DM, and AP), we randomly permuted case/control status at 20,000 times and repeated the differential expression analysis (logFC between two randomly assigned group) using limma, yielding a null distribution consisting of 20000 of Spearman's correlation coefficients. The permutated p-value was determined as follows:

$$P - value = \frac{\text{\# of cases (Correlation coefficient of permuted status > Correlation coefficient of actual status)}}{\text{20000 (iteration time), a null distribution}}$$

## Hyper-parameters for rWGCNA (For 2.5 section in Manuscript, Fig.S1, and Fig.S2)

A robust version of Weighted Gene Co-Expression Network Analysis (rWGCNA) [11, 12] was used to minimize the influence of potential outlier samples with the biweight midcorrelation (bicor). For

the co-expression network, a signed network was used, with a soft-threshold power of 9 to achieve approximate scale-free topology ($R^2 > 0.8$, Fig.S1). The network dendrogram was created by using average linkage hierarchical clustering of the topological overlap dissimilarity matrix (1 − TOM). Modules were defined as branches of the dendrogram using the hybrid dynamic tree-cutting method [13]. We set the pamStage as "False". We iterated construction of co-expression network at 40 times as setting different hyper-parameters, and selected a case with consisting modules that were conserved during different setting with ratio of 0.25. In other words, all modules in the selected case were curated at more than 10 times among 40 simulations. Finally, we determined a consensus quantile threshold, a minimum module size, deepsplit, and pamStage as 0.1, 100, and 1, respectively.

## Measurement of differential connectivity (For 2.5 section)

The differential connectivity was measured as following steps.

Step 1) Given gene i and gene j, we calculated $k_{ij} = |cor(i, j)|^{\beta}$, with defining k, cor (), and $\beta$ as connectivity, biweight midcorrelation, and hyper-parameter (named as soft-threshold power) determined by researcher, respectively.

Step 2) We measured the mean connectivity (MC) of gene i with other genes in a module consisting of N genes, as $MC_i = \sum_{j=1}^{N} k_{ij(i \neq j)}$. Then, using RNA expression data of disease samples, we measured MCs of N genes ($MC_{1\ N}^{Disease(Dx)} = MC_1^{Dx}, MC_2^{Dx}, MC_3^{Dx}, \cdots, MC_N^{Dx}$), and using those of control samples, we also calculate MC of N genes ($MC_{1\ N}^{Control}$). We compared $MC_{1\ N}^{Disease}$ and $MC_{1\ N}^{Control}$ using t-test, and selected a module with Bonferroni-corrected p-value < 0.05, and named the module as the module with modular differential connectivity (MDC).

## Preprocessing of brain and fat RNA expression datasets (For 3.7 section in Manuscript)

We downloaded GSE5281 and GSE20966 dataset from the GEO DB [5]. Then we scaled and normalized the two datasets via log transformation and quantile normalization, respectively. We mapped probe ID to Entrez ID, and removed probes without a matched-Entrez ID. Afterward, we excluded approximately 30% of the probes that had small variance across samples. For multiple probes mapped with a gene (Entrez ID), we selected the probe with the maximum average expression value via 'collapseRows' function in WGCNA package [8].

## References


1. Zhou, X., et al., Human symptoms-disease network. Nat Commun, 2014. 5: p. 4212.

2. Menche, J., et al., Disease networks. Uncovering disease-disease relationships through the incomplete interactome. Science, 2015. 347(6224): p. 1257601.

3. Kim, J., J.J. Kim, and H. Lee, An analysis of disease-gene relationship from Medline abstracts by DigSee. Sci Rep, 2017. 7: p. 40154.

4. Irizarry, R.A., et al., Exploration, normalization, and summaries of high density oligonucleotide array probe level data. Biostatistics, 2003. 4(2): p. 249-264.

5. Clough, E. and T. Barrett, The Gene Expression Omnibus Database. Methods Mol Biol, 2016. 1418: p. 93-110.

6. Smyth, G.K., Limma: linear models for microarray data, in Bioinformatics and computational biology solutions using R and Bioconductor. 2005, Springer. p. 397-420.

7. Berger, J.A., et al., Optimized LOWESS normalization parameter selection for DNA microarray data. BMC bioinformatics, 2004. 5(1): p. 194.

8. Langfelder, P. and S. Horvath, WGCNA: an R package for weighted correlation network analysis. BMC bioinformatics, 2008. 9(1): p. 559.

9. Kanehisa, M. and S. Goto, KEGG: kyoto encyclopedia of genes and genomes. Nucleic Acids Res, 2000. 28(1): p. 27-30.

10. Liberzon, A., et al., The Molecular Signatures Database (MSigDB) hallmark gene set collection. Cell Syst, 2015. 1(6): p. 417-425.

11. Parikshak, N.N., et al., Integrative functional genomic analyses implicate specific molecular pathways and circuits in autism. Cell, 2013. 155(5): p. 1008-1021.

12. Gandal, M.J., et al., Shared molecular neuropathology across major psychiatric disorders parallels polygenic overlap. Science, 2018. 359(6376): p. 693-697.

13. Langfelder, P., B. Zhang, and S. Horvath, Defining clusters from a hierarchical cluster tree: the Dynamic Tree Cut package for R. Bioinformatics, 2007. 24(5): p. 719-720.

**Supplementary Figure S1.** Soft Threshold Power (Hyper-parameter) of Co-Expression Network (rWGCNA). rWGCNA, robust version of Weighted Gene Co-Expression Network Analysis

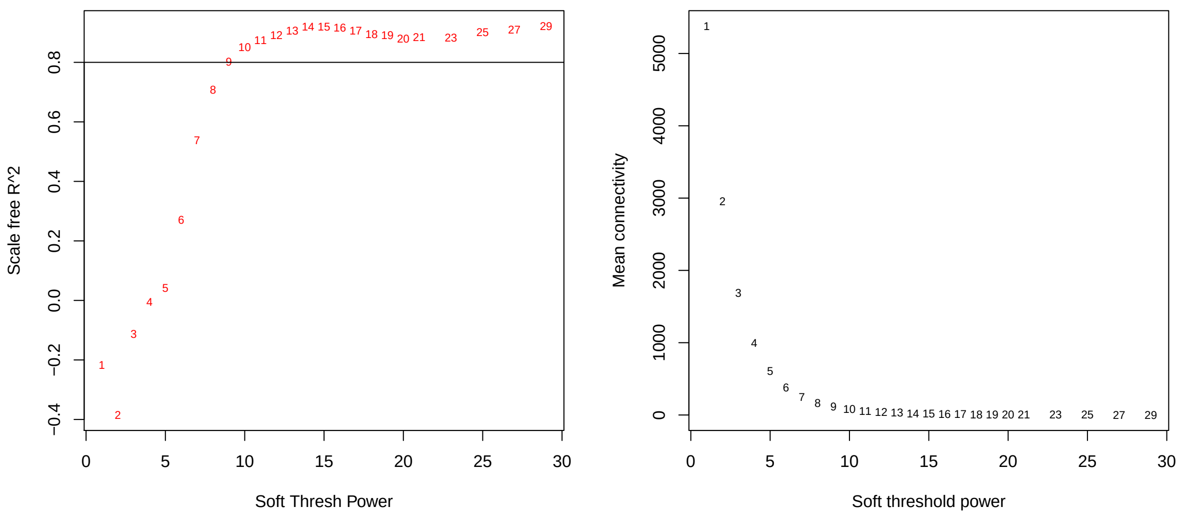

**Supplementary Figure S2.** DS, MMS, DCOR, PAM (Hyper-parameters) of Co-Expression Network (rWGCNA). DS, deepsplit; MMS, minimum module size; DCOR, consensus quantile threshold, PAM, pamStage; rWGCNA, robust version of Weighted Gene Co-Expression Network Analysis

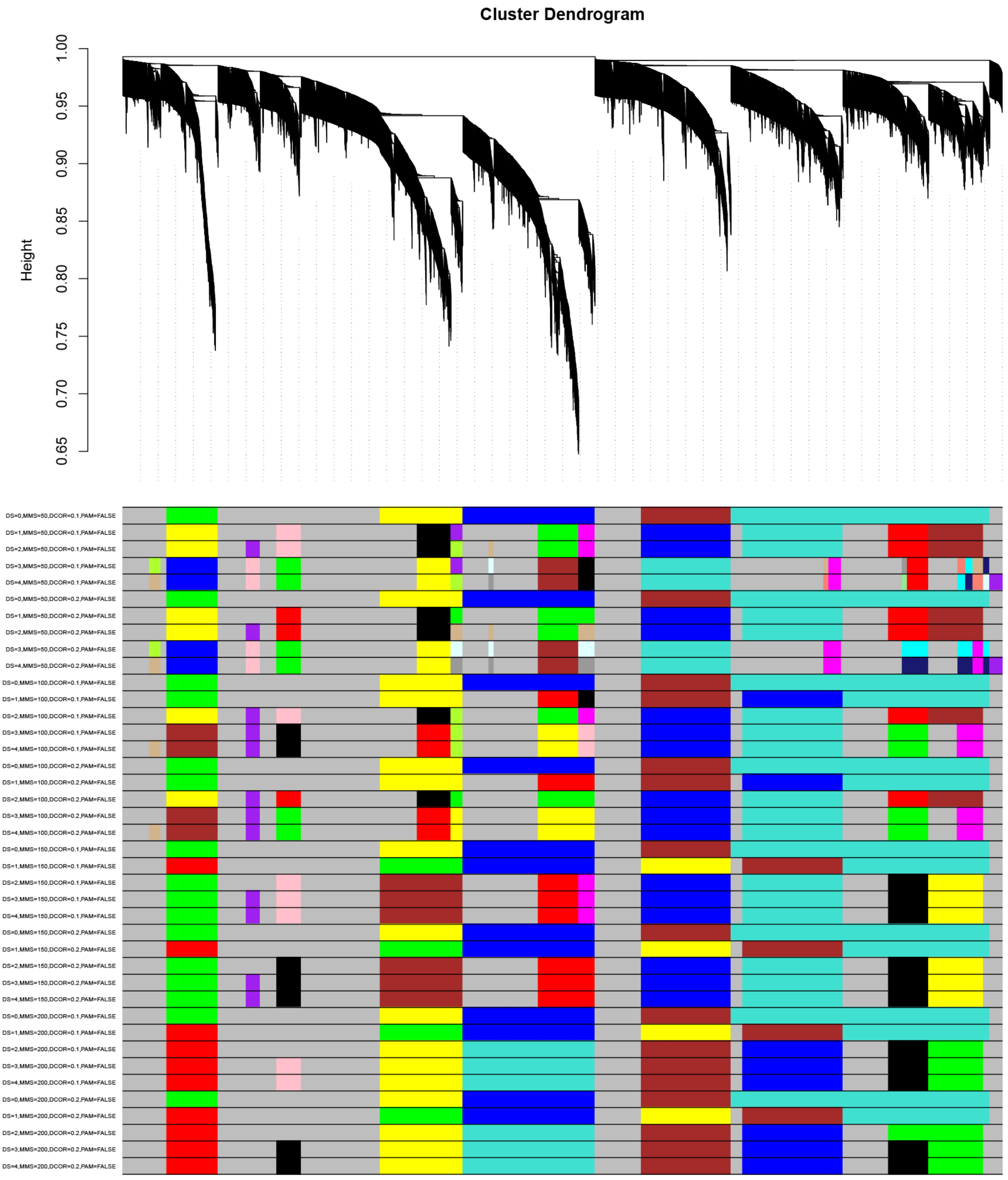

**Supplementary Figure S3.** Correlation matrices from Spearman correlation for all pairwise compar-isons among blood datasets in each disease. AD, Alzheimer's Disease; DM, diabetes mellitus

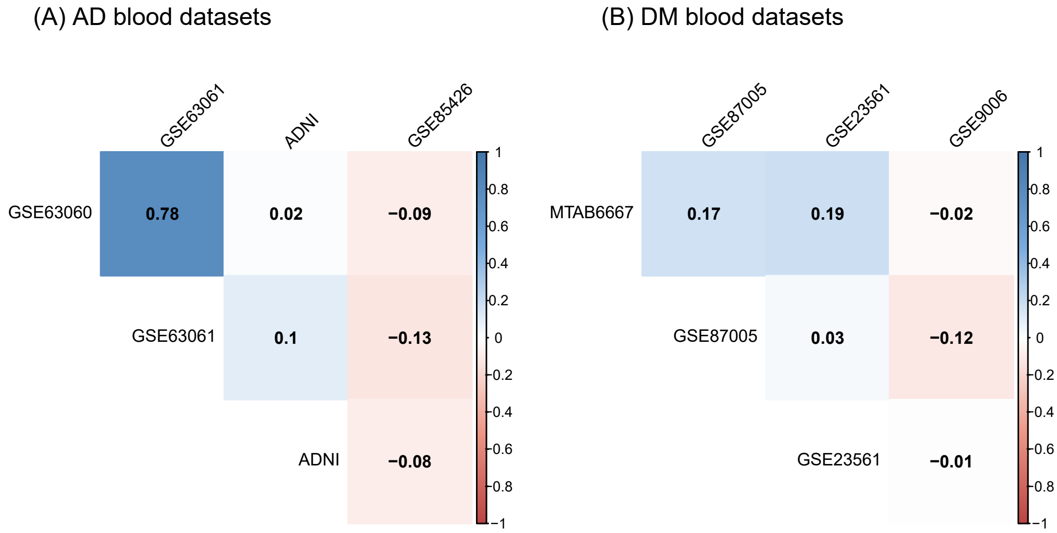

**Supplementary Figure S4.** Comparison of gene expressions between integrated AD blood and inte-grated DM blood datasets.

We integrated ADNI, GSE63060, GSE63061, and GSE85426 into one large AD dataset, and E-MTABL-6667, GSE87005, and GSE23561 into one large DM dataset. The number of genes that is commonly present in the seven gene expression datasets were 1,116.

AD, Alzheimer's Disease; DM, diabetes mellitus; SCC, Spearman's correlation coefficient, FC, fold change

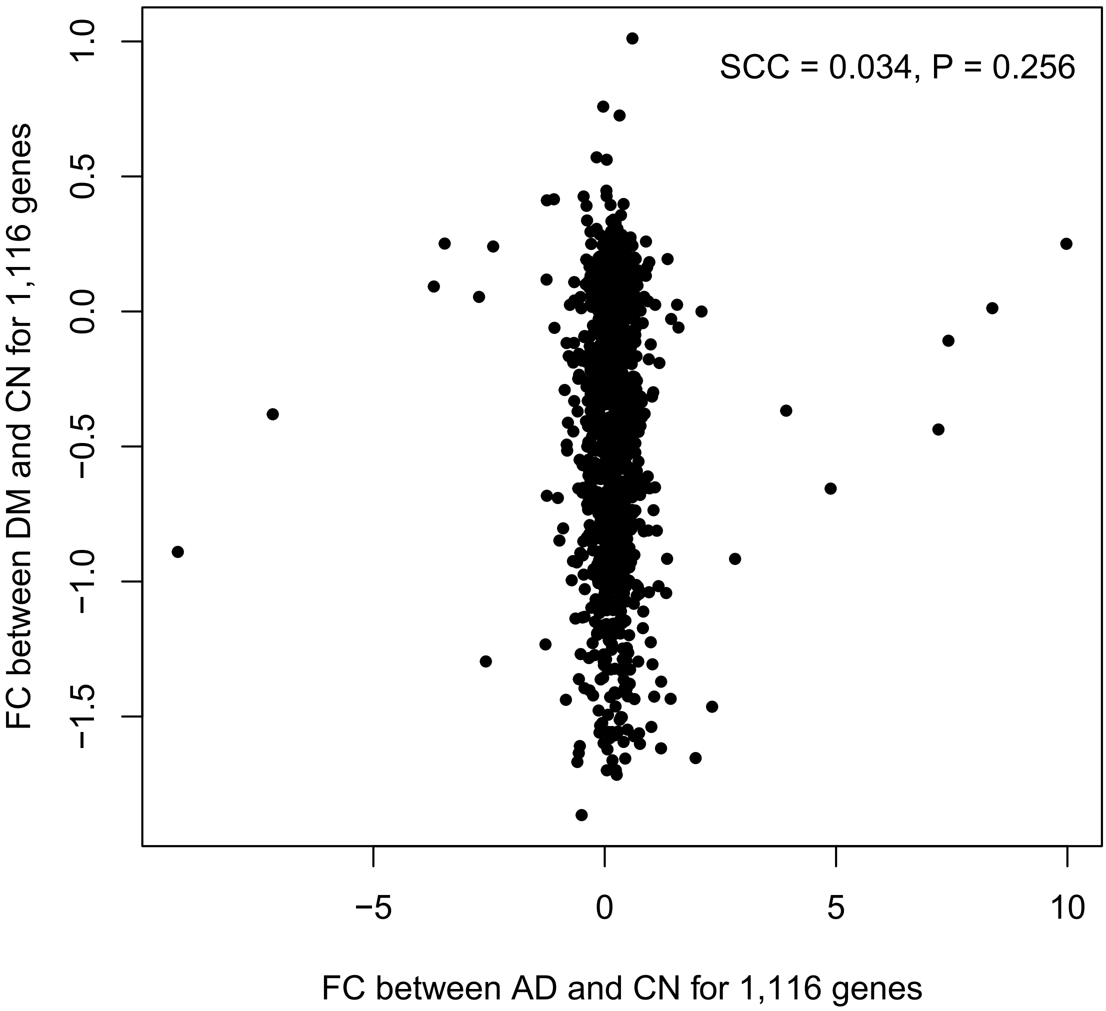

**Supplementary Table S1.** All analyzed datasets (AD and DM)

| AD datasets | | | | |
|---|---|---|---|---|
| | # of controls | # of patients | Platform | Tissue type |
| GSE63060 | 104 | 145 | Illumina HumanHT-12 V3.0 expression beadchip (GPL6947) | blood |
| GSE63061 | 134 | 139 | Illumina HumanHT-12 V4.0 expression beadchip (GPL10558) | blood |
| GSE85426 | 90 | 90 | Agilent-028004 SurePrint G3 Human GE 8x60K Microarray (GPL14550) | blood |
| ADNI | 136 | 63 | Affymetrix Human Genome U219 Array | blood |
| GSE5281 | 74 | 87 | Affymetrix Human Genome U133 Plus 2.0 Array (GPL570) | brain |
| DM datasets | | | | |
| | # of control | # of patients | Platform | Tissue type |
| E-MTAB-6667 | 40 | 19 | Illumina HumanHT-12 v4.0 (A-MEXP-2072) | blood (used), colon (not-used) |
| GSE87005 | 20 | 20 | Agilent-014850 Whole Human Genome Microarray 4x44K G4112F (GPL6480) | blood |
| GSE23561 | 9 | 8 | Human 50K Exonic Evidence-Based Oligonucleotide array (GPL10775) | blood |
| GSE9006 | 24 | 12 | Affymetrix Human Genome U133B Array (GPL97) | blood |
| GSE81608 | 6 (651 cells) | 12 (919 cells) | Illumina HiSeq 2500 (Homo sapiens) | pancreas (single-cell) |
| GSE20966 | 10 | 10 | Affymetrix Human X3P Array | pancreas |

AD, Alzheimer’s disease; DM, diabetes mellitus
Words in brackets (Platform column) indicate datasets consisting of probes or probe-sets ID and Entrez ID.

**Supplementary Table S2.** Enriched pathways of genes in green module

| GOID | Term | Count | FDR |
|---|---|---|---|
| GO:0006412 | translation | 74 | <0.001 |
| GO:0043043 | peptide biosynthetic process | 74 | <0.001 |
| GO:0022613 | ribonucleoprotein complex biogenesis | 62 | <0.001 |
| GO:0006614 | SRP-dependent cotranslational protein targeting to membrane | 32 | <0.001 |
| GO:0043604 | amide biosynthetic process | 74 | <0.001 |
| GO:0006613 | cotranslational protein targeting to membrane | 32 | <0.001 |
| GO:0042254 | ribosome biogenesis | 51 | <0.001 |
| GO:0045047 | protein targeting to ER | 32 | <0.001 |
| GO:0072599 | establishment of protein localization to endoplasmic reticulum | 32 | <0.001 |
| GO:0006518 | peptide metabolic process | 76 | <0.001 |
| GO:0000184 | nuclear-transcribed mRNA catabolic process, nonsense-mediated decay | 33 | <0.001 |
| GO:0016071 | mRNA metabolic process | 68 | <0.001 |
| GO:0006413 | translational initiation | 38 | <0.001 |
| GO:0043603 | cellular amide metabolic process | 82 | <0.001 |
| GO:0006396 | RNA processing | 78 | <0.001 |
| GO:0070972 | protein localization to endoplasmic reticulum | 32 | <0.001 |
| GO:0034641 | cellular nitrogen compound metabolic process | 248 | <0.001 |
| GO:0000956 | nuclear-transcribed mRNA catabolic process | 38 | <0.001 |
| GO:0016072 | rRNA metabolic process | 43 | <0.001 |
| GO:0006364 | rRNA processing | 42 | <0.001 |
| GO:0006402 | mRNA catabolic process | 38 | <0.001 |
| GO:1901564 | organonitrogen compound metabolic process | 124 | <0.001 |
| GO:0006807 | nitrogen compound metabolic process | 254 | <0.001 |
| GO:0006401 | RNA catabolic process | 39 | <0.001 |
| GO:1901566 | organonitrogen compound biosynthetic process | 92 | <0.001 |
| GO:0006612 | protein targeting to membrane | 34 | <0.001 |
| GO:0019080 | viral gene expression | 34 | <0.001 |
| GO:0019083 | viral transcription | 33 | <0.001 |
| GO:0044033 | multi-organism metabolic process | 34 | <0.001 |
| GO:0010467 | gene expression | 205 | <0.001 |
| GO:1902582 | single-organism intracellular transport | 59 | <0.001 |
| GO:0034470 | ncRNA processing | 45 | <0.001 |
| GO:0034660 | ncRNA metabolic process | 53 | <0.001 |
| GO:0072594 | establishment of protein localization to organelle | 57 | <0.001 |
| GO:0034655 | nucleobase-containing compound catabolic process | 42 | <0.001 |
| GO:0070125 | mitochondrial translational elongation | 22 | <0.001 |
| GO:0006415 | translational termination | 23 | <0.001 |
| GO:0070126 | mitochondrial translational termination | 22 | <0.001 |
| GO:0019439 | aromatic compound catabolic process | 44 | <0.001 |
| GO:0046700 | heterocycle catabolic process | 43 | <0.001 |
| GO:0006414 | translational elongation | 26 | <0.001 |
| GO:0044265 | cellular macromolecule catabolic process | 68 | <0.001 |
| GO:0033365 | protein localization to organelle | 65 | <0.001 |
| GO:0044270 | cellular nitrogen compound catabolic process | 43 | <0.001 |
| GO:0044237 | cellular metabolic process | 311 | <0.001 |
| GO:0006605 | protein targeting | 55 | <0.001 |
| GO:0046907 | intracellular transport | 89 | <0.001 |
| GO:1901361 | organic cyclic compound catabolic process | 43 | <0.001 |
| GO:0032543 | mitochondrial translation | 23 | <0.001 |
| GO:0090150 | establishment of protein localization to membrane | 38 | <0.001 |
| GO:0046483 | heterocycle metabolic process | 210 | <0.001 |
| GO:0006139 | nucleobase-containing compound metabolic process | 206 | <0.001 |
| GO:0006725 | cellular aromatic compound metabolic process | 210 | <0.001 |
| GO:0006886 | intracellular protein transport | 65 | <0.001 |
| GO:1901360 | organic cyclic compound metabolic process | 214 | <0.001 |
| GO:0019058 | viral life cycle | 40 | <0.001 |
| GO:0008152 | metabolic process | 321 | <0.001 |
| GO:0009057 | macromolecule catabolic process | 68 | <0.001 |
| GO:0007005 | mitochondrion organization | 47 | <0.001 |
| GO:0043624 | cellular protein complex disassembly | 25 | <0.001 |
| GO:0016032 | viral process | 59 | <0.001 |
| GO:0044260 | cellular macromolecule metabolic process | 261 | <0.001 |
| GO:0044764 | multi-organism cellular process | 59 | <0.001 |
| GO:0044267 | cellular protein metabolic process | 176 | <0.001 |
| GO:0044238 | primary metabolic process | 301 | <0.001 |

**Supplementary Table S2.** Enriched pathways of genes in green module, continued

| GOID | Term | Count | FDR |
|---|---|---|---|
| GO:0051649 | establishment of localization in cell | 93 | <0.001 |
| GO:0034613 | cellular protein localization | 80 | <0.001 |
| GO:0044419 | interspecies interaction between organisms | 59 | <0.001 |
| GO:0044403 | symbiosis, encompassing mutualism through parasitism | 59 | <0.001 |
| GO:0044085 | cellular component biogenesis | 120 | <0.001 |
| GO:0070727 | cellular macromolecule localization | 80 | <0.001 |
| GO:0044248 | cellular catabolic process | 80 | <0.001 |
| GO:0044271 | cellular nitrogen compound biosynthetic process | 175 | <0.001 |
| GO:0043933 | macromolecular complex subunit organization | 106 | <0.001 |
| GO:0072657 | protein localization to membrane | 38 | <0.001 |
| GO:0071704 | organic substance metabolic process | 308 | <0.001 |
| GO:0022904 | respiratory electron transport chain | 18 | <0.001 |
| GO:0000375 | RNA splicing, via transesterification reactions | 29 | <0.001 |
| GO:0051641 | cellular localization | 107 | <0.001 |
| GO:1902580 | single-organism cellular localization | 61 | <0.001 |
| GO:0022900 | electron transport chain | 18 | <0.001 |
| GO:0044249 | cellular biosynthetic process | 202 | <0.001 |
| GO:0043241 | protein complex disassembly | 26 | <0.001 |
| GO:0015031 | protein transport | 85 | <0.001 |
| GO:0045184 | establishment of protein localization | 90 | <0.001 |
| GO:0032984 | macromolecular complex disassembly | 26 | <0.001 |
| GO:0042775 | mitochondrial ATP synthesis coupled electron transport | 16 | <0.001 |
| GO:0042773 | ATP synthesis coupled electron transport | 16 | <0.001 |
| GO:0009205 | purine ribonucleoside triphosphate metabolic process | 26 | <0.001 |
| GO:0042273 | ribosomal large subunit biogenesis | 14 | <0.001 |
| GO:0006119 | oxidative phosphorylation | 17 | <0.001 |
| GO:0022618 | ribonucleoprotein complex assembly | 23 | <0.001 |
| GO:0008380 | RNA splicing | 32 | <0.001 |
| GO:0009199 | ribonucleoside triphosphate metabolic process | 26 | <0.001 |
| GO:0009144 | purine nucleoside triphosphate metabolic process | 26 | <0.001 |
| GO:0009058 | biosynthetic process | 204 | <0.001 |
| GO:1902600 | hydrogen ion transmembrane transport | 17 | <0.001 |
| GO:0000398 | mRNA splicing, via spliceosome | 27 | <0.001 |
| GO:0000377 | RNA splicing, via transesterification reactions with bulged adenosine as nucleophile | 27 | <0.001 |
| GO:0071826 | ribonucleoprotein complex subunit organization | 23 | <0.001 |
| GO:0046034 | ATP metabolic process | 24 | <0.001 |
| GO:0015992 | proton transport | 19 | <0.001 |
| GO:0090304 | nucleic acid metabolic process | 173 | <0.001 |
| GO:0006818 | hydrogen transport | 19 | <0.001 |
| GO:0042278 | purine nucleoside metabolic process | 29 | <0.001 |
| GO:0034645 | cellular macromolecule biosynthetic process | 169 | <0.001 |
| GO:0019538 | protein metabolic process | 180 | <0.001 |
| GO:0009141 | nucleoside triphosphate metabolic process | 26 | <0.001 |
| GO:1901657 | glycosyl compound metabolic process | 32 | <0.001 |
| GO:0009116 | nucleoside metabolic process | 31 | <0.001 |
| GO:0034622 | cellular macromolecular complex assembly | 53 | <0.001 |
| GO:0008104 | protein localization | 98 | <0.001 |
| GO:0043170 | macromolecule metabolic process | 266 | <0.001 |
| GO:0009167 | purine ribonucleoside monophosphate metabolic process | 25 | <0.001 |
| GO:0009126 | purine nucleoside monophosphate metabolic process | 25 | <0.001 |
| GO:0016070 | RNA metabolic process | 158 | <0.001 |
| GO:0006397 | mRNA processing | 33 | <0.001 |
| GO:0051436 | negative regulation of ubiquitin-protein ligase activity involved in mitotic cell cycle | 13 | <0.001 |
| GO:0046128 | purine ribonucleoside metabolic process | 28 | <0.001 |
| GO:0009059 | macromolecule biosynthetic process | 171 | <0.001 |
| GO:0051439 | regulation of ubiquitin-protein ligase activity involved in mitotic cell cycle | 13 | <0.001 |
| GO:1904667 | negative regulation of ubiquitin protein ligase activity | 13 | <0.001 |
| GO:0042255 | ribosome assembly | 12 | <0.001 |
| GO:0045333 | cellular respiration | 19 | <0.001 |
| GO:0009161 | ribonucleoside monophosphate metabolic process | 25 | <0.001 |
| GO:0051437 | positive regulation of ubiquitin-protein ligase activity involved in regulation of mitotic cell cycle transition | 13 | <0.001 |
| GO:1901576 | organic substance biosynthetic process | 196 | 0.001 |
| GO:0031145 | anaphase-promoting complex-dependent catabolic process | 13 | 0.001 |
| GO:0071840 | cellular component organization or biogenesis | 196 | 0.001 |
| GO:0051444 | negative regulation of ubiquitin-protein transferase activity | 13 | 0.001 |

**Supplementary Table S2.** Enriched pathways of genes in green module, continued

| GOID | Term | Count | FDR |
|---|---|---|---|
| GO:0009123 | nucleoside monophosphate metabolic process | 25 | 0.001 |
| GO:1901575 | organic substance catabolic process | 79 | 0.001 |
| GO:0009119 | ribonucleoside metabolic process | 28 | 0.001 |
| GO:1904668 | positive regulation of ubiquitin protein ligase activity | 13 | 0.001 |
| GO:0015980 | energy derivation by oxidation of organic compounds | 23 | 0.001 |
| GO:2000058 | regulation of protein ubiquitination involved in ubiquitin-dependent protein catabolic process | 14 | 0.002 |
| GO:0061024 | membrane organization | 51 | 0.002 |
| GO:1904666 | regulation of ubiquitin protein ligase activity | 13 | 0.002 |
| GO:0051443 | positive regulation of ubiquitin-protein transferase activity | 14 | 0.002 |
| GO:0022411 | cellular component disassembly | 32 | 0.002 |
| GO:0006091 | generation of precursor metabolites and energy | 27 | 0.003 |
| GO:0044802 | single-organism membrane organization | 45 | 0.003 |
| GO:0009056 | catabolic process | 81 | 0.003 |
| GO:2000060 | positive regulation of protein ubiquitination involved in ubiquitin-dependent protein catabolic process | 13 | 0.003 |
| GO:0009987 | cellular process | 386 | 0.005 |
| GO:0033036 | macromolecule localization | 103 | 0.005 |
| GO:0006120 | mitochondrial electron transport, NADH to ubiquinone | 10 | 0.008 |
| GO:0071822 | protein complex subunit organization | 69 | 0.01 |
| GO:0051438 | regulation of ubiquitin-protein transferase activity | 14 | 0.01 |
| GO:0042776 | mitochondrial ATP synthesis coupled proton transport | 7 | 0.015 |
| GO:0016043 | cellular component organization | 186 | 0.016 |
| GO:0031397 | negative regulation of protein ubiquitination | 14 | 0.024 |
| GO:0007346 | regulation of mitotic cell cycle | 29 | 0.032 |
| GO:0032981 | mitochondrial respiratory chain complex I assembly | 10 | 0.033 |
| GO:0010257 | NADH dehydrogenase complex assembly | 10 | 0.033 |
| GO:0097031 | mitochondrial respiratory chain complex I biogenesis | 10 | 0.033 |
| GO:1901990 | regulation of mitotic cell cycle phase transition | 22 | 0.033 |
| GO:0071702 | organic substance transport | 95 | 0.035 |
| GO:0065003 | macromolecular complex assembly | 68 | 0.036 |
| GO:1903322 | positive regulation of protein modification by small protein conjugation or removal | 17 | 0.038 |
| GO:0006996 | organelle organization | 124 | 0.045 |
| GO:0030529 | intracellular ribonucleoprotein complex | 102 | <0.001 |
| GO:1990904 | ribonucleoprotein complex | 102 | <0.001 |
| GO:0044391 | ribosomal subunit | 55 | <0.001 |
| GO:0005840 | ribosome | 59 | <0.001 |
| GO:0032991 | macromolecular complex | 234 | <0.001 |
| GO:0044446 | intracellular organelle part | 309 | <0.001 |
| GO:0044422 | organelle part | 310 | <0.001 |
| GO:0015934 | large ribosomal subunit | 37 | <0.001 |
| GO:0022626 | cytosolic ribosome | 36 | <0.001 |
| GO:0043229 | intracellular organelle | 373 | <0.001 |
| GO:0043227 | membrane-bounded organelle | 374 | <0.001 |
| GO:0043231 | intracellular membrane-bounded organelle | 353 | <0.001 |
| GO:0070013 | intracellular organelle lumen | 196 | <0.001 |
| GO:0044424 | intracellular part | 397 | <0.001 |
| GO:0031974 | membrane-enclosed lumen | 198 | <0.001 |
| GO:0043233 | organelle lumen | 196 | <0.001 |
| GO:0005622 | intracellular | 400 | <0.001 |
| GO:0005740 | mitochondrial envelope | 69 | <0.001 |
| GO:0031966 | mitochondrial membrane | 67 | <0.001 |
| GO:0005743 | mitochondrial inner membrane | 57 | <0.001 |
| GO:0043226 | organelle | 384 | <0.001 |
| GO:0019866 | organelle inner membrane | 59 | <0.001 |
| GO:0044445 | cytosolic part | 40 | <0.001 |
| GO:0044444 | cytoplasmic part | 282 | <0.001 |
| GO:0005739 | mitochondrion | 105 | <0.001 |
| GO:0044429 | mitochondrial part | 77 | <0.001 |
| GO:0022625 | cytosolic large ribosomal subunit | 23 | <0.001 |
| GO:0031981 | nuclear lumen | 161 | <0.001 |
| GO:0044428 | nuclear part | 169 | <0.001 |
| GO:0005737 | cytoplasm | 330 | <0.001 |
| GO:0031967 | organelle envelope | 77 | <0.001 |
| GO:0031975 | envelope | 77 | <0.001 |
| GO:0005829 | cytosol | 148 | <0.001 |
| GO:0000313 | organellar ribosome | 21 | <0.001 |

**Supplementary Table S2.** Enriched pathways of genes in green module, continued

| GOID | Term | Count | FDR |
|---|---|---|---|
| GO:0005761 | mitochondrial ribosome | 21 | <0.001 |
| GO:0005634 | nucleus | 240 | <0.001 |
| GO:0043228 | non-membrane-bounded organelle | 162 | <0.001 |
| GO:0043232 | intracellular non-membrane-bounded organelle | 162 | <0.001 |
| GO:0044455 | mitochondrial membrane part | 29 | <0.001 |
| GO:0015935 | small ribosomal subunit | 19 | <0.001 |
| GO:0098800 | inner mitochondrial membrane protein complex | 24 | <0.001 |
| GO:0098798 | mitochondrial protein complex | 26 | <0.001 |
| GO:0005654 | nucleoplasm | 126 | <0.001 |
| GO:0005762 | mitochondrial large ribosomal subunit | 14 | <0.001 |
| GO:0000315 | organellar large ribosomal subunit | 14 | <0.001 |
| GO:0005730 | nucleolus | 55 | <0.001 |
| GO:0005759 | mitochondrial matrix | 36 | <0.001 |
| GO:0043234 | protein complex | 153 | <0.001 |
| GO:0070469 | respiratory chain | 17 | <0.001 |
| GO:0022627 | cytosolic small ribosomal subunit | 13 | <0.001 |
| GO:0016469 | proton-transporting two-sector ATPase complex | 13 | <0.001 |
| GO:0005746 | mitochondrial respiratory chain | 16 | <0.001 |
| GO:0071013 | catalytic step 2 spliceosome | 16 | <0.001 |
| GO:0098803 | respiratory chain complex | 15 | <0.001 |
| GO:0031090 | organelle membrane | 106 | <0.001 |
| GO:0070062 | extracellular exosome | 109 | <0.001 |
| GO:1903561 | extracellular vesicle | 109 | <0.001 |
| GO:0043230 | extracellular organelle | 109 | <0.001 |
| GO:0005753 | mitochondrial proton-transporting ATP synthase complex | 9 | <0.001 |
| GO:0045259 | proton-transporting ATP synthase complex | 9 | <0.001 |
| GO:0005681 | spliceosomal complex | 19 | <0.001 |
| GO:1902494 | catalytic complex | 54 | <0.001 |
| GO:0044464 | cell part | 405 | 0.001 |
| GO:0030532 | small nuclear ribonucleoprotein complex | 11 | 0.001 |
| GO:0005623 | cell | 405 | 0.001 |
| GO:0097525 | spliceosomal snRNP complex | 10 | 0.004 |
| GO:0005747 | mitochondrial respiratory chain complex I | 9 | 0.011 |
| GO:0045271 | respiratory chain complex I | 9 | 0.011 |
| GO:0030964 | NADH dehydrogenase complex | 9 | 0.011 |
| GO:0005763 | mitochondrial small ribosomal subunit | 7 | 0.013 |
| GO:0000314 | organellar small ribosomal subunit | 7 | 0.013 |
| GO:0000502 | proteasome complex | 10 | 0.014 |
| GO:1990234 | transferase complex | 37 | 0.014 |
| GO:1990204 | oxidoreductase complex | 12 | 0.027 |
| GO:0031988 | membrane-bounded vesicle | 117 | 0.037 |
| GO:0003735 | structural constituent of ribosome | 50 | <0.001 |
| GO:0003723 | RNA binding | 122 | <0.001 |
| GO:0044822 | poly(A) RNA binding | 99 | <0.001 |
| GO:0005198 | structural molecule activity | 55 | <0.001 |
| GO:0003676 | nucleic acid binding | 146 | 0.001 |
| GO:0015078 | hydrogen ion transmembrane transporter activity | 16 | 0.001 |
| GO:0050136 | NADH dehydrogenase (quinone) activity | 10 | 0.014 |
| GO:0003954 | NADH dehydrogenase activity | 10 | 0.014 |
| GO:0008137 | NADH dehydrogenase (ubiquinone) activity | 10 | 0.014 |
| GO:0016655 | oxidoreductase activity, acting on NAD(P)H, quinone or similar compound as acceptor | 11 | 0.014 |
| hsa03010 | Ribosome | 43 | <0.001 |
| hsa00190 | Oxidative phosphorylation | 28 | <0.001 |
| hsa05010 | Alzheimer's disease | 22 | <0.001 |
| hsa05012 | Parkinson's disease | 20 | <0.001 |
| hsa05016 | Huntington's disease | 21 | 0.003 |
| hsa03040 | Spliceosome | 17 | 0.004 |
| TF | RPS27A, MTERF3, RPL7, SSB, RIOK2, PHF5A, GTF2F2, CEBPG, ZMAT2, PUM3, LARP7, ZNF17, POLE4, ZNF613, ZNF26, CGGBP1, ZNF22, ZNF302, PSMD10, BCLAF1, ZNF277, S100A8, THAP12, GPBP1, PAX7, CEBPZ, ZNF816, COPS4, GTF2B, GTF2E2, COMMD6, SUB1, PSMA6, TCEAL8, ZBED5, MYNN, SLC30A9, TAX1BP1, CNBP, ZC3H15, BOLA2 | NA | |
